# The Epigenetic Effects of Coffee

**DOI:** 10.3390/molecules28041770

**Published:** 2023-02-13

**Authors:** Qi Ding, Yan-Ming Xu, Andy T. Y. Lau

**Affiliations:** Laboratory of Cancer Biology and Epigenetics, Department of Cell Biology and Genetics, Shantou University Medical College, Shantou 515041, China

**Keywords:** coffee extract, caffeine, chlorogenic acid, caffeic acid, epigenetic effects, diseases

## Abstract

In this review, we discuss the recent knowledge regarding the epigenetic effects of coffee extract and the three essential active ingredients in coffee (caffeine, chlorogenic acid, and caffeic acid). As a popular beverage, coffee has many active ingredients which have a variety of biological functions such as insulin sensitization, improvement of sugar metabolism, antidiabetic properties, and liver protection. However, recent researches have shown that coffee is not only beneficial for human, but also bad, which may be due to its complex components. Studies suggest that coffee extract and its components can potentially impact gene expression via alteration of DNA methylation, histone modifications, and ncRNA expression; thus, exert long lasting impacts on the epigenome. More importantly, coffee consumption during pregnancy has been linked to multiple negative effects on offspring due to epigenetic modifications; on the other hand, it has also been linked to improvements in many diseases, including cancer. Therefore, understanding more about the epigenetic effects associated with coffee components is crucial to finding ways for improving human health.

## 1. Introduction

**Figure 1:**
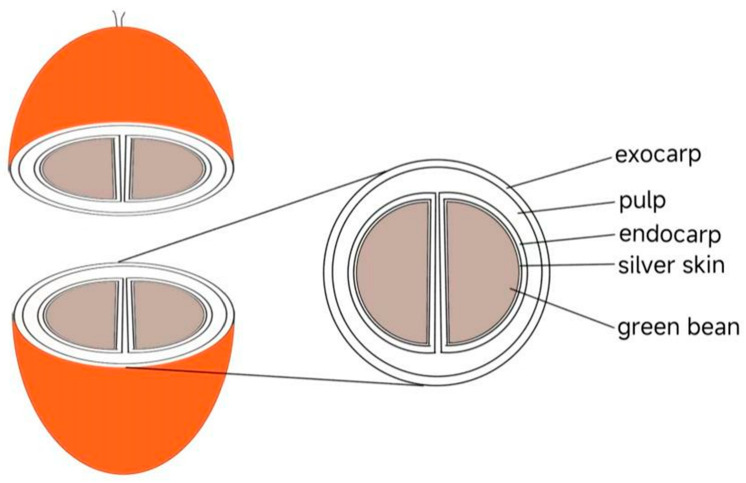
The basic structure of a coffee cherry.

**Figure 2:**
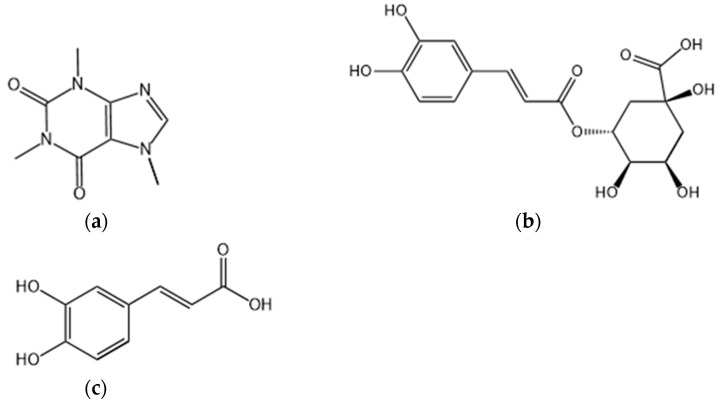
The chemical structure of the three coffee ingredients: (**a**) caffeine, IUPAC name: 1,3,7-trimethylpurine-2,6-dione; (**b**) chlorogenic acid, IUPAC name: (1S,3R,4R,5R)-3-[(*E*)-3-(3,4-dihydroxyphenyl)prop-2-enoyl]oxy-1,4,5-trihydroxycyclohexane-1-carboxylic acid; (**c**) caffeic acid, IUPAC name: (*E*)-3-(3,4-dihydroxyphenyl)prop-2-enoic acid.

As one of the popular beverages in modern times, coffee is being consumed widely by humans. The production of coffee consists of multistep processes and it all started with the raw material—the beans from coffee cherries (Figure 1). According to the National Coffee Association of U.S.A., coffee consumption in the U.S.A. is at a two-decade high. Americans drink an estimated 491 million cups of coffee per day, and 66% of Americans drank coffee in the past day, which exceeded bottled water consumption [1]. A recent study showed that coffee has many biological functions such as lowering blood sugar, protecting the liver and nerves due to diverse chemical components in coffee, including alkaloids, phenolic acids, flavonoids, and terpenoids [2]. Furthermore, the content of active ingredients in coffee is influenced by many factors, including coffee variety, origin, extraction procedure, roasting temperature, and grinding degree [3,4,5]. However, as a double-edged sword, coffee is both good and bad for the human body. On the one hand, benefits of coffee consumption were seen in Alzheimer’s disease (AD), nonalcoholic fatty liver disease (NAFLD), cardiovascular disease (CVD), and some cancers (including hepatocellular cancer and possibly breast cancer) [6,7,8,9,10]. On the other hand, coffee contributes to the increased risk of higher blood pressure [11] and declined physical functioning [12]. From this, we can speculate that both the positive and negative effects may spring from different compounds within coffee. Although a variety of review articles on coffee’s effect are available in the literature, yet, a recent update on its epigenetic effects is lacking. Therefore, we put our focus here on the epigenetic effects of coffee extract and the three essential active ingredients in coffee: caffeine (CAF), chlorogenic acid (CGA), and caffeic acid (CA or CaA) (Figure 2).

## 2. Epigenetics and Diseases

Epigenetics refers to molecular modifications on chromatin that can regulate gene activity, are independent of DNA sequence, and are mitotically stable. There are three primary epigenetic regulations that have been well studied: DNA methylation, histone modifications, and noncoding RNAs (ncRNAs) [13]. In the past decade, the epigenetic effects of coffee ingredients have been revealed, whereby coffee is able to impact the epigenome by modulating DNA methylation/demethylation, histone modifications, and ncRNA expression (Table 1), and potentially linked to the development of various human pathological conditions. In the next sections, the latest knowledge regarding the epigenetic effects of coffee on various diseases are discussed.

## 3. The Impact of Coffee during Pregnancy and in Diseases

### 3.1. Impact during Pregnancy

CAF consumption during pregnancy has been reported to be associated with intrauterine growth retardation (IUGR), low birth weight, subfertility, and spontaneous abortion. With regard to the long-term health of the offspring, prenatal CAF exposure (PCE) also affects the long-term development of the fetus and elicits adult diseases, including increasing the risk of childhood acute lymphoblastic leukemia [14]. Here, the latest knowledge regarding the epigenetic effects of coffee components is discussed in PCE offspring. In rats, most of the apparent modification effects are due to the increased synthesis of maternal adrenal endogenous corticosterone promoted by CAF, while a high concentration of maternal glucocorticoid (GC) can suppress the production of fetal adrenal endogenous corticosterone. Considering that GC plays an important role in offspring development, PCE predisposes offspring to a range of diseases.

#### 3.1.1. Impact on Hypothalamic–Pituitary–Adrenal (HPA) Axis

Studies have shown that PCE affects fetal adrenal GC (cortisol in humans and corticosterone in rodents) synthesis through methylation and affects the development of the HPA axis, ultimately leading to IUGR in offspring mice, as well as high susceptibility to adult metabolic syndrome. 

In 2012, in primary fetal rat hippocampal neuron, it was found that CAF treatment enhanced the total methylation ratio at nt −358 to −77 of the promoter of 11β-hydroxysteroid dehydrogenase-2 (11β-HSD-2) and then inhibited its expression. A decrease in 11β-HSD-2 may be related to the increased expression of 11β-hydroxysteroid dehydrogenase 1 (11β-HSD-1) and glucocorticoid receptor (GR) in the fetal hippocampus, eventually leading to the reduced corticotrophin-releasing hormone (CRH). Considering that the hippocampus is an important regulatory center of HPA axis, as well as the most sensitive and vulnerable neural target site for circulatory GC, PCE inhibited the development of fetal HPA axis and further retarded fetal development [15]. In the same year, in NCI-H295A cells, a CpG site demethylation at nt −682 of the steroidogenic acute regulatory protein (StAR) promoter was found after CAF treatment, which was maintained over 10 passages after terminating CAF treatment and induced long-term stimulation of StAR expression. As the initial and rate-limiting step in adrenocortical steroid biosynthesis, the increased expression of StAR promoted cortisol production [16]. However, in 2014, using rats as the model, Ping et al. found that PCE suppressed the function of corticosterone synthesis in the fetal adrenal. This study showed that CAF could notably increase the total methylation rate and alter histone acetylation status in fetal adrenal steroidogenic factor-1 (SF-1) promoter, which decreased the SF-1 expression. SF-1 could regulate steroidogenic enzyme expression, including StAR, 3β-hydroxysteroid dehydrogenase, and cytochrome P450 cholesterol side-chain cleavage (P450scc, P450c21, and P450c11), so inhibiting the expression of SF-1 reduced the expression of the steroidogenic enzymes and further inhibited fetal adrenal steroidogenesis, which contributed to HPA axis dysfunction [17]. In 2021, using rats as the model, He et al. also found that PCE could inhibit the adrenal StAR expression by regulating the H19/let-7 axis in the offspring. The study showed that PCE led to hypermethylation of H19 and its low expression in fetal adrenal gland, which was maintained to adulthood, thus enhancing the inhibitory effect of GR/let-7 pathway on adrenal StAR expression by weakening the “molecular sponge” effect. In addition, the methylation levels of H19 increased in the F1 and F3 rats, but decreased in the F2 rats, which may be related to the changing serum corticosterone level, indicating the multigeneration inheritance of adrenal StAR expression inhibition [18].

In addition to regulating steroidogenic enzyme expression, studies exist showing PCE can suppress the cholesterol uptake to inhibit the production of steroid hormones. In 2015, using rats as the model, it was found that prenatal CAF ingestion could induce DNA hypermethylation in scavenger receptor class B type I (SR-BI) promoter and inhibit SR-BI expression in fetal adrenal gland, which subsequently suppressed the cholesterol uptake, followed by decreasing the production of steroid hormones and thus increasing IUGR rates [19].

#### 3.1.2. Impact on Bone

Studies have shown that PCE affected bone development through epigenetic modifications. In 2012, using rats as the model, it was found that CAF exposure decreased histone methylation (H3K36me2, H3K4me2, and H3K4me3) of fetal insulin-like growth factor 1 (IGF-1) in the liver, which was responsible for the reduced levels of IGF-1 in fetuses. In addition, the suppressed expression of IGF-1 was also demonstrated in growth plates. IGF-1 is an important mediator of muscle and bone growth, so the inhibition of systemic and local IGF-1 levels retarded chondrogenesis and skeletal growth [20]. In 2018, again using rats as the model, the same group found a new epigenetic modification of IGF-1 induced by PCE in female offspring’s cartilage, which increased susceptibility to osteoarthritis. It was found that PCE decreased H3K9 and H3K27 acetylation at the IGF-1 promoter region via overexposure to corticosterone, which impeded the expression of IGF-1 signaling pathway aggrecan and COL2A1 genes, thus reducing the synthesis of extracellular matrix in cartilage, and ultimately leading to delayed development of articular cartilage [21]. Similarly, in 2020, Li et al. found that PCE inhibited the function of cartilage matrix synthesis in male adult offspring rats through consistently decreasing H3K9ac and the expression levels of transforming growth factor beta receptor 1 (TGFβR1). It then blocked the TGFβ signaling pathway in fetal cartilage and induced susceptibility to osteoarthritis [22].

In addition to affecting extracellular matrix synthesis in articular chondrocytes, PCE can affect bone differentiation through changing the intrauterine epigenetic modifications, thereby affecting osteogenic development. In 2019, using rats as the model, it was found that PCE decreased the DNA methylation level at the promoter region of bone angiotensin-converting enzyme (ACE) gene, which increased the expression of ACE. The increased ACE further participated in the constant activation of bone renin angiotensin system (RAS), which played a vital role in bone development and contributed to suppressing the osteogenic differentiation of bone marrow mesenchymal stem cells (BMSCs), thus causing a decrease in body length and bone mineral content of offspring rats [23]. Similarly, in 2020, using rats as the model, it was found that PCE suppressed osteogenic differentiation and lowered peak bone mass through decreasing the H3K9ac level of 11β-HSD2 in the bone tissue, which was maintained in the adult male offspring. However, these changes were not evident in the adult female offspring, suggesting gender differences in PCE [24].

#### 3.1.3. Impact on Ovary

Studies have shown that PCE affected ovarian development through histone modification. In 2021, using rats as the model, it was found that H3K27ac level at the IGF-1 promoter region and the expression of IGF-1 were decreased by PCE in the fetal ovary, which inhibited the expression of steroidogenic enzymes and lowered estradiol synthesis accompanied by abnormal ovarian development in the fetus. In addition, intrauterine programming is also the main pathophysiological basis for early-stage ovarian catch-up growth in PCE offspring linked with hypothalamic–pituitary–ovarian axis activity and ovarian dysfunction in adulthood [25].

#### 3.1.4. Impact on Cardiovascular Development

Studies have shown that PCE affected heart development through altering DNA modification and regulating ncRNA expression. In 2014, using rats as the model to detect the influence of A1AR and CAF in adult offspring left ventricle, it was found that in the veh+/+ vs. caff+/+ comparison, PCE greatly changed DNA methylation patterns within the genomic DNA. The differentially methylated regions included promoter regions, primary transcripts, known CpG islands or miRNA promoter regions which were related to the development of hearts. Moreover, significant differentially methylation was found in 103 miRNA regions in the ventricle [26]. In addition to altering methylation status in adult offspring ventricles, PCE alters DNA methylation levels and the expression of ncRNA in embryonic heart ventricle, which affects the offspring’s heart development. In 2014, Fang et al. found in vitro and in vivo that CAF altered the expression of some microRNAs and significantly increased the global cardiac DNA methylation in embryonic heart ventricles, which contributed to altering the expression of important cardiac structural and hormonal genes and also changed the cardiac morphology related to cardiovascular development and diseases [27].

The research findings from the above studies suggest that PCE has a long-term epigenetic effect in rats, being highly toxic to bone, ovary, cardiovascular development, and HPA axis development, as well as kidney, liver, and cancer development, which are outlined below, leading to a variety of related diseases linked to moderate coffee consumption during pregnancy. In addition, data from clinical studies show that CAF consumption may increase the risk of pregnancy loss, low birth weight, childhood obesity, childhood acute leukemia, autism spectrum disorder in offspring, suggesting that even consumption of the amount of coffee containing an acceptable daily dose of CAF in some studies turned out to be unsafe [28]. However, another study revealed that CAF’s effects on pregnancy outcomes was highly variable across individuals in both rodents and humans [14].

### 3.2. Impact on Cancer

From an umbrella review of meta-analyses and an overview about coffee consumption and cancer risk, it was shown that there was highly suggestive evidence for an inverse association between coffee intake and risk of liver, endometrial, and breast cancer among postmenopausal women. With respect to the association with other organs, including the esophagus, pancreas, colorectum, kidneys, bladder, ovaries, and prostate, the results were less clear [8,29].

#### 3.2.1. Colon Carcinoma

Studies have shown that coffee could improve colon carcinoma through regulating ncRNA expression. In 2017, using Caco-2 human colon carcinoma cells as the model, it was found in vitro that coffee extract reduced the expression of KRAS by activating two miRNAs, miR-30c and miR-96. Thus, coffee suppressed the KRAS signaling pathways including the mitogen-activated protein kinase (MAPK) and phosphatidylinositol 3-kinase (PI3K) signaling pathways and further exerted an inhibitory effect on cell proliferation [30]. In 2021, Luque-Badillo et al. found in vitro that one of the coffee active ingredients, CGA, could decrease colon cancer cell proliferation by inhibiting the expression of the miR-31 oncogene which played a vital role in promoting cell-cycle progression [31].

In 2022, using 1,2-dimethylhydrazine/deoxycholic acid (DMH/DCA)-induced colon carcinogenesis rats as the model, it was found in vivo that CAF + CGA intervention changed the expression of six miRNAs in the colon. Of those miRNAs, the DMH/DCA group and CAF + CGA intervention group shared the differential expression of onco miR miR-21a-5p, which was involved in “negative regulation of the ERK1 and ERK2 cascade”, “negative regulation of the TGF-β receptor signaling pathway”, and “positive regulation of the apoptotic process”. These processes were related to the decreased epithelial cell proliferation, the increased apoptosis in colonic crypts, and also the decreased levels of proinflammatory cytokines IL-6, IL-17, and TNF-α in the colon, which helped to attenuate the development of preneoplastic aberrant crypt foci (ACF) [32].

The results from the above studies provide insightful perspectives of CAF and/or CGA’s epigenetic impacts on miRNAs, suggesting their essential role in preventing the colon carcinoma development and as a chemotherapeutic agent.

#### 3.2.2. Leukemia

In 2019, in a subpopulation of the Avon Longitudinal Study of Parents and Children (ALSPAC) cohort, it was found in cord blood at birth that the intake of maternal sugary caffeinated drinks and coffee consumption resulted in larger proportions of hypermethylated compared to hypomethylated CpGs (73% and 60%, respectively). Moreover, the concordance of the change in direction of methylation influenced by exposure and in acute lymphoblastic leukemia (ALL) was high for sugary caffeinated drink intake (over 70%), but low for coffee consumption suggesting something other than CAF driving the larger number of concordant changes identified for sugary caffeinated drink intake [33].

In 2020, it was found in vitro that CGA decreased global DNA methylation in human acute T-cell leukemia cells (Jurkat), but not in human promyelocytic leukemia cells (HL-60), suggesting that the mechanisms via which CGA acts could be cell-type specific and the hypomethylation function of CGA is beneficial for improving hematological malignancies whose pathogenic processes involve DNA methylation damage [34].

#### 3.2.3. Breast Cancer

Study has indicated a new action role of CaA or CGA as an agent to reverse aberrant DNA methylation, as well as its involvement in attenuating the development of breast cancer. In 2006, it was found that treatment of cultured human breast cancer cells with CaA or CGA caused a concentration-dependent inhibition of DNA methylation at the promoter region of the RARb gene [35].

#### 3.2.4. Cancer Risk

Studies have suggested that CaA can improve the cancer stem cell (CSC)-like properties. In 2015, Li et al. found in vitro and in vivo that CaA reduced DNA methylation of miR-148a promoter, which improved the expression of miR-148a. Then, miR-148a targeted the SMAD2-3′UTR, leading to a decreased expression of SMAD2, which inhibited TGFβ/SMAD2 signaling and attenuated the cancer stem cell (CSC)-like properties, suggesting a new approach for the treatment of human cancers [36].

In addition, another study showed that coffee can reduce the cancer risk induced by carcinogens. In 2022, it was found coffee extract significant decreased the expression of miR-9-3 in the liver, the expression of miR-9-3 and miR-134 in the spleen, the expression of miR-9-3, miR-124-1, and miR-134 in the kidney, which were elevated by 7,12-dimethylbenz[a]anthracene (DMBA) carcinogen; the inhibition of these miRNAs could be antioxidant and anti-inflammatory, in addition to blocking some DMBA-activated factors including oncogenes (RAS and MYC) while inducing the expression of PTEN and SIRT tumor suppressor genes. Therefore, this study showed the chemopreventive effects of coffee in cancer risk [37].

The data from the above studies suggest the potential epigenetic effects of coffee ingredients and their essential role in preventing cancers, including colon carcinoma, breast cancer, and leukemia development.

### 3.3. Cataract and Glaucoma

In 2022, it was genetically predicted that coffee consumption showed a suggestive association with senile cataract [38]. In two studies, it was found that higher coffee consumption was associated with a higher risk of primary open-angle glaucoma (POAG) and open-angle glaucoma (OAG) compared with those who did not drink coffee [39,40]. In addition, a large prospective study revealed a positive association of heavier coffee consumption with risk of exfoliation glaucoma or exfoliation glaucoma suspect (EG/EGS) [41]. However, in some cross-sectional studies, it was shown that habitual CAF consumption was not associated with the risk of glaucoma development [42,43,44]. The epigenetic effects of coffee ingredients in cataract and glaucoma are listed below, showing that CAF benefits cataracts and CGA protects against glaucoma.

In 2013, to detect the protective effect of CAF in lenses against oxidative stress induced by a high-galactose diet, Varma et al. found that CAF reduced the toxic miRNA transcription in lenses from the rats fed by galactose and CAF, compared with the galactose alone group. As these miRNAs are known to induce apoptosis and cell death by gene silencing, this study suggests CAF’s possible pharmacological use in cataract prevention [45]. In 2019, another ingredient of coffee, CGA, was demonstrated to promote lncRNA-TUG1 expression, which positively regulated nuclear factor erythroid-2 related factor 2 (Nrf2) expression, and thus, decreased H_2_O_2_-induced retinal ganglion cell (RGC) apoptosis and ROS level, as well as increased RGCs viability in vitro, suggesting that CGA could prevent oxidative stress injury and protect against glaucoma [46].

### 3.4. Pathogen Infection

The below-described studies show that CAF or CGA regulates the inflammatory response or promotes autophagy through epigenetic effects to reduce the damage caused by pathogens to organisms.

In 2016, Li et al. found that CAF inhibited the expression of miR-301b when treated with lipopolysaccharide (LPS) or *Pseudomonas aeruginosa* in vitro and in vivo. A decrease in miR-301b, thus, increased c-Myb expression, which positively modulated anti-inflammatory cytokines IL-4 and TGF-β1 but negatively regulated proinflammatory cytokines MIP-1α and IL-17A, leading to a subdued inflammatory response. Therefore, CAF could prevent mice from suffering severe organ injury, including the lung, liver, and kidney injury. In addition, the repression of miR-301b also resulted in elevated levels of neutrophil infiltration in lung, which may have caused stronger bacterial clearance at early times, thereby alleviating infectious symptoms in mice [47]. Similarly, in 2022, Liu et al. found that CGA alleviated pathological damage in lung tissues from LPS-induced acute lung injury (ALI) mice. This study found that CGA heightened the expression of miR-223, which may have targeted the inhibition of nucleotide-binding oligomerization domain-like receptor protein 3 (NLRP3) expression, ultimately reducing the LPS-induced inflammatory response in ALI mice [48].

In addition, CGA can inhibit schistosomiasis-induced liver fibrosis and improve *Salmonella Typhimurium* (ST) infection in intestinal tissue. In 2017, Wang et al. found that CGA inhibited the expression of miR-21 and increased the level of Smad7 in vitro and in vivo. Through regulating IL-13/miR-21/Smad7 signaling interactions, CGA inhibited schistosomiasis-induced liver fibrosis [49]. In 2020, it was found in vitro and in vivo that CGA increased the expression of lncRNA-GAS5, which competitively bound to miR-23a to upregulate PTEN and inhibit the downstream p38 MAPK pathway after ST infection. Through regulating the lncRNA-GAS5/miR-23a/PTEN axis and the p38 MAPK pathway, which promote autophagy in ST infection, CGA alleviated ST infection in intestinal tissue [50].

### 3.5. Impact on Brain

A growing number of studies suggest that coffee is beneficial to neurodegenerative diseases, including AD and Parkinson’s disease (PD) [51]. For example, in 2019, one study showed that higher lifetime coffee intake may contribute to lowering the risk of AD or related cognitive decline by reducing pathological cerebral amyloid deposition [52]. However, it is difficult to find studies that report the epigenetic mechanism of coffee in relation to these diseases. A list of the epigenetic effects of coffee ingredients on the brain is provided below.

In 2019, using ethanol-drinking (UChB) rats as the model, Rossetto et al. found that CAF could restore the expression of some miRNAs in the cerebellum and plasma, which was changed by ethanol. Considering that ethanol predisposes to an inflammatory process by altering the expression of miRNAs, this study suggested that CAF acted as a neuroprotector agent against ethanol for the cerebellar tissue [53]. In addition to alcohol, another component of coffee, CGA, was found to reduce the damage caused by hypoxic ischemia reperfusion in brain cells. In 2022, using hypoxic ischemia reperfusion cells as the model, Fan et al. found that CGA upregulated MIR497HG which targeted and repressed miR-29b-3p expression, and then upregulated sirtuin-1 (SIRT1) to initiate the SIRT1/nuclear factor-kappa B (NF-κB) signaling pathway, leading to a decrease in inflammation, oxidative stress, and neuron apoptosis elicited by oxygen and glucose deprivation (OGD) in cells [54].

Moreover, in 2020, Hu et al. found that CaA may be a valuable therapeutic option for major depressive disorder (MDD). Using the chronic unpredictable mild stress (CUMS) rats, there was an increase in 5mC/5hmC at the promoter region of Bdnf and Comt gene in the hippocampus and a decrease in 5mC/5hmC at the promoter region of Comt gene in the prefrontal cortex in the model group, which may be restored by CaA and, therefore, influenced their expressions. Thus, CaA exerted a slight antidepressant-like effect [55].

The results from the above studies provide insightful perspectives into the epigenetic impacts of three coffee ingredients, suggesting their potential role in protecting against MDD and protecting the nerves from the effects of alcohol and OGD.

### 3.6. Impact on Liver

Consumption of coffee has been presented to benefit liver health, including hepatitis B and C, NAFLD and alcoholic liver disease [56].

Studies have shown that some components of coffee can alter the epigenetic modifications of the genes involved in lipid metabolism in the liver to improve some related diseases. In 2011, it was found that treatment of Hepa 1–6 cells with coffee polyphenols (CPP) significantly increased miR-122, which played an important role in regulating lipid metabolism. CPP could regulate the expression of sterol regulatory element-binding protein SREBP-1c and the downstream targets FASN and ACC in hepatocytes, which enhanced energy metabolism and reduced lipogenesis, thus leading to the suppression of body fat accumulation in CPP-treated mice with the high-fat diet [57]. In 2019, using PCE rats as the model, it was found that H3K14ac and H3K27ac levels, as well as the expression of lipogenic genes (SREBP-1c and FASN), were increased by PCE-induced high GC levels through inhibiting SIRT1 expression. So, PCE contributed to increasing triglyceride synthesis and abnormal hepatic lipid accumulation in female offspring, which continued throughout postnatal and adult life and increased the susceptibility to adult NAFLD [58].

In both male and female offsprings, PCE caused symptoms of hypercholesterolemia by changing the epigenetic modifications of some genes in the liver. In 2018, in PCE female offspring rats, it was found that the levels of hepatic H3K9ac and H3K14ac were increased at the promoter of 3-hydroxy-3-methylglutaryl-CoA reductase (HMGCR), which enhanced hepatic cholesterol synthesis, and thus increased the cholesterol levels in blood. In addition, the resultant catch-up growth after birth also had effects on hepatic cholesterol metabolism in female offspring. Lastly, PCE induced hypercholesterolemia in adult offspring [59]. In 2019, it was found that PCE increased histone acetylation and expression levels of hepatic cholesterol synthesis-related genes (Srebf2, Hmgcr, and Hmgcs1) in male offspring during intrauterine life, which continued throughout adulthood and elevated hepatic cholesterol synthesis, resulting in hypercholesterolemia in the male offspring rats [60].

In addition, in 2017, Yang et al. found that CGA inhibited liver fibrosis induced by CCl_4_. This study showed that CGA decreased the expression of miR-21, inhibited the levels of TGF-β1, and elevated the expression of Smad7 in vitro and in vivo. Perhaps through the regulation of the miR-21-regulated TGF-β1/Smad7 signaling, CGA suppressed the CCl_4_-induced liver fibrosis, which suggested that CGA might be a new anti-fibrosis agent to improve liver fibrosis [61].

The data from the above studies suggest the potential epigenetic effects of coffee ingredients and their essential role in preventing liver fibrosis and body fat accumulation, and they also revealed the adverse effects of PCE on liver development because of the epigenetic effects of CAF.

### 3.7. Impact on Kidney

Studies showed that coffee is beneficial for some kidney-related diseases. In a systematic review and meta-analysis study, it was shown that a higher CAF intake may be associated with a lower risk of kidney stones [62]. In another systematic review and meta-analysis, it was revealed that coffee consumption may be associated with a lower risk of chronic kidney disease (CKD) [63]. In 2022, a meta-analysis of the published cohort evidence was suggestive of an inverse association between coffee consumption and renal cancer risk [64]. At the same year, from the Atherosclerosis Risk in Communities (ARIC) study, it was shown that higher coffee intake was associated with a lower risk of acute kidney injury (AKI) [65]. Two studies of the epigenetic effects of coffee ingredients on the kidney are described below.

Some studies have shown that CaA is beneficial to diabetic nephropathy (DN) by regulating the expression of autophagy-related genes through epigenetic modification. In 2017, using high-fat diet and streptozotocin-induced (HFD-STZ) diabetic male rats as the model, it was found that treatment with CaA suppressed the expression of miR-133b, -342, and -30a expression in the diabetic kidney, which was linked to autophagy gene upregulation [RB 1-inducible coiled coil protein (RB1CC1), microtubule-associated proteins 1A/1B light chain 3 (MAP1LC3B), and autophagy-related gene (ATG-12)], subsequently causing the restoration of autophagy activity which contributed to improving DN [66]. Similarly, in 2019, using STZ-induced DN rats as the model, it was found that CaA also exhibited protective action against DN via downregulating miR-636 expression in the kidney, which was associated with promotion of autophagy [67].

In 2019, using rats as the model, Zhu et al. found that PCE can also affect the function of kidney. It was shown that PCE reduced the H3K9ac level at the KLF4 promoter region in fetal podocyte, which contributed to the low expressional programming of KLF4, followed by the reduced expression of genes nephrin, Wilms’ tumor 1, and podocin in male offspring kidney, thus resulting in podocyte developmental toxicity and the loss of podocytes [68].

The data from the above studies provide insightful perspectives of CaA’s epigenetic impacts and suggest its potential role in improving DN, while also reveal the adverse effects of PCE on kidney development.

### 3.8. Impact on Human Genome

Methylation studies of the human genome have suggested that coffee consumption may be beneficial for several diseases, including neurodegenerative diseases and liver diseases. In 2017, a study demonstrated that habitual coffee consumption could alter DNA methylation sites in human genome. It revealed alterations in methylation status of CpG sites located near 11 genes including GPR132, BSCL2, MALRD1, GRK5, PSMD8, FSTL5, and PTHLH. Moreover, it was found that the top-ranked CpGs appeared to be linked to genes involved in lipid metabolism and immune response. Furthermore, many differentially methylated CpGs were located in/near the genes reported to be associated with coffee-related chronic diseases or the common neurodegenerative diseases (PD and AD), for which coffee consumption had been suggested to be protective [69]. Recently, epigenome-wide association studies (EWAS) meta-analysis of coffee consumption has revealed 11 CpGs surpassing the epigenome-wide significance threshold in peripheral blood-derived DNA, which were annotated to the AHRR, F2RL3, FLJ43663, HDAC4, GFI1, and PHGDH genes; pathway analysis of these genes showed enrichment for serine biosynthesis, xenobiotic metabolism signaling and association with inflammatory response. These studies indicate that coffee-associated changes in DNA methylation levels may explain the mechanism of action of coffee consumption in conferring risk of some diseases including liver disease [70].

### 3.9. Other Epigenetic Effects

In addition to specific diseases, studies of epigenetic modifications of coffee toward specific genes involved in various physiological activities are described below.

In 2008, Mukwevho et al. found the mechanism via which CAF increased the expression of GLUT4 glucose transporter in C_2_C_12_ myotubes. This study suggested that CAF treatment could increase the acetylation of histone H3 at the myocyte enhancer factor 2 (MEF2) site on the GLUT4 gene and increase the binding of MEF2A to the site in C_2_C_12_ myotubes, contributing to the increased expression of GLUT4 [71]. In 2015, Shi et al. found that, in HeLa cells, CAF could affect post-transcriptional regulation by increasing the splicing factor SRSF2 levels. This study showed that ~8% of the miRNAs (especially miR-183-5p and miR-33a-5p) examined (99 of ~1200) were found to be downregulated after CAF treatment, which contributed to the changed translation efficiency of SRSF2 transcripts and inhibited nonsense-mediated decay (NMD), which would break the negative feedback loop controlling SRSF2 gene expression. Therefore, CAF caused a surge of SRSF2 protein levels and influenced alternative splicing choices in SRSF2-targeted gene [72]. 

Studies have suggested the protective effects of CAF against ethanol and carcinogens DMBA. In 2019, using UChB rats as the model, it was found that the ingestion of CAF was capable of reducing the levels of serum miRNAs, miR-9-3p, -15b-5p, -16-5p, and -222-3p, which were increased in UchB mice. The reversed expression of these miRNAs is related to many diseases and plays a vital role in organic systems, especially in the central nervous system, emphasizing that CAF had a protective effect in the presence of ethanol [73]. In 2021, using DMBA-treated mice, it was found that carcinogen DMBA induced significant repetitive long interspersed element-1 (LINE-1) DNA hypomethylation in the liver, spleen, and kidney, while coffee significantly prevented the hypomethylating effects. The LINE-1 DNA methylation pattern plays a key role in both carcinogenesis and chemoprevention. Therefore, coffee showed chemopreventive effects [74].

Moreover, in pigs and bees, studies have revealed some methylation effects of CAF. In 2008, using pigs as the model, it was found that nuclear remodeling of somatic cell nuclear transfer embryos (NTs) could be regulated by CAF. This study showed that CAF affected the methylation status of NTs, which was related to nuclear reprogramming, while CAF also increased the maturation promoting factor (MPF) activity which contributed to inducing a high rate of premature chromosome condensation (PCC) and lowered apoptotic cell rate of NT blastocyst. Thus, the in vitro development of embryos was influenced [75]. In 2014, using honeybees as the model, it was found that CAF slowed the global DNA methylation levels during aging and had great metabolic effects on honeybees, which helped in extending the lifespan and increasing resistance to Nosema [76]. 

More detailed information about the epigenetic modifications caused by coffee or coffee components is listed in Table 2.

## 4. Conclusions

Among the three essential bioactive components examined in coffee, it is amazing that each of them exerts distinct epigenetic effects that ultimately modulate gene expressions and plays a vital role in various diseases, suggesting the benefits of coffee to some people, but not everyone (especially pregnant women). From recent studies, we can know that the effect of coffee on people is related to the amount of coffee consumed, gender, and personal constitution. For example, in 2014, a review revealed that some people were reported experiencing irregular heartbeat or headaches; thus, they were reluctant to drink coffee, suggesting individual variation to coffee intolerance [77]. In 2021, one study found that, compared with those who consumed <1 cup/day of coffee, when men consumed 1 and ≥2 cups/day, an association of a lower risk of hearing impairment existed, but this was not seen in women [78]. Moreover, in 2022, a study found that high coffee consumption was associated with smaller total brain volumes and increased odds of dementia [79], which suggested avoiding heavy coffee intake. However, from the above discussion, it can be seen that coffee extracts and their active ingredients also counteract cancer (including colon carcinoma, leukemia, and breast cancer), cataract and glaucoma, some brain diseases, pathogen infection, liver fibrosis, DN, and so on. Given the benefits of coffee’s active ingredients, combining them with drugs could have great potential in the treatment of various diseases. For example, Bagdas et al. revealed that CGA and its active metabolites, alone or in combination with other bioactive extracts, could be used as a complementary and alternative medicine for the treatment of arthritis [80].

As a popular drink and a complex mixture which contains many bioactive ingredients, the importance of coffee to human health has been well recognized for many years. Therefore, further understanding about the epigenetic effects associated with coffee is crucial to finding ways for improving human health. 

## Figures and Tables

**Table 1:** An overview of the epigenetic effects of coffee components.

Coffee Ingredients	DNA Modification	Histone Modification	ncRNA Expression
Coffee extract	Yes	No data	Yes
Caffeine	Yes	Yes	Yes
Chlorogenic acid	Yes	No data	Yes
Caffeic acid	Yes	No data	Yes

**Table 2:** Coffee-induced epigenetic alterations.

Focus of Study	Model	Impact	Gene Modified/Epigenetic Alterations	Disease Involved/Consequence	Ref
HPA axis	Primary fetal rat hippocampal neurons were treated with 300 µM CAF for 24 h, and examined using refseq promoter methylation array	DNA	A total of 1183 genes were hypermethylated and 496 genes were hypomethylated Hypermethylation was observed at nt −358 to −77 of the 11β-HSD-2 promoter	Inhibited the functional development of the fetal HPA axis	[15]
	Primary fetal rat hippocampal neurons were treated with CAF (0, 3, 30, and 300 µM) for 24 h, and examined using bisulfite sequencing PCR		Dose-dependently increased the total methylation ratio within nt −358 to −77 of the 11β-HSD-2 promoter 30 µM of CAF increased the methylation at nt −220, −214, −158, and −152, and 300 µM of CAF increased the methylation at nt −220, −214, −211, −193, −167, −152, −130, and −111 significantly		
	Human adrenocortical cell line NCI-H295A was treated with 4 μM or 40 μM CAF for 48 h	DNA	Resulted in a substantially decreased frequency of methylation of a single CpG site at nt −682 of the StAR promoter, but few effects on the total methylation status of the promoter	Increased the cortisol production in cells	[16]
	From gestational day (GD) 11 to GD20, the pregnant Wistar rats were intragastrically treated with 120 mg/kg CAF once a day	DNA and Histone	Among the 36 CpG sites of the SF-1 promoter, nt −162, −139, −108, −104, −91, −53, −50, and −14 showed higher frequency of single CpG methylation, which was associated with the increased expression of Dnmt1, Dnmt3b, and Dnmt3a The acetylation level of H3K9 and H3K14 was decreased at the SF-1 promoter which was associated with elevated expression levels of both HDAC1 and HDAC2	Led to the occurrence of IUGR	[17]
	From GD9 to GD20, pregnant rats in the experimental group were given 120 mg/kg of CAF by gavage once a day	DNA and ncRNA	The total methylation rate of fetal adrenal H19 promoter region was increased due to the reduced CTCF expression and the increased DNMT3a/3b expression induced by high level of GC through activating GR Increased adrenal miRNA let-7c expression in F1 female fetal rats induced by high level of GC through activating GR	Resulted in adrenal dysfunction	[18]
	From GD7 to GD17, the rats in the CAF group were intragastrically treated with 60 mg/kg CAF at 8:00 a.m. once a day (40% dose on GD7, 80% dose on GD8, full dosage since GD9)	DNA	CAF markedly increased the total methylation rate of SR-BI promoter in fetal adrenal. Among the 55 CpG sites, nt −522, −504, −396, −359, −343, −320, −255, −165, −123, −115, −71, −40, −27, −6, 3, 73, and 99 showed increased frequency of single-CpG methylation after CAF treatment	Suppressed the cholesterol uptake followed by the decreased production of steroid hormones and increased IUGR rates	[19]
Bone	From GD11 to GD20, CAF was administered intragastrically to the pregnant Wistar rats at a dose of 120 mg/kg per day	DNA	H3K36me2, H3K4me2, and H3K4me3 at P2 and 3-UTR of IGF-1 genes were reduced in the fetal liver due to the high levels of GC in utero caused by CAF	Contributed to the retardation of both chondrogenesis and skeletal growth	[20]
	From GD9 to GD20, the pregnant Wistar rats were intragastrically treated with 30, 60, and 120 mg/kg/day CAF	Histone	The H3K9ac level of TGFβR1 was reduced in the fetal cartilage via the increased fetal serum corticosterone concentration that promoted GR activation and recruited HDAC2 to decrease the H3K9ac	Induced cartilage dysplasia and increased osteoarthritis susceptibility in male adult offspring	[22]
	From GD9 to GD20, CAF was administered to the pregnant rats by gavage at a dose of 12 mg/100 g body weight per day	DNA	CAF decreased the frequency of DNA methylation at the promoter of ACE in the bone tissue of the offspring related to the increased GR-C/EBPα signaling in the functioning of GC	PCE caused fetal growth retardation and adult osteopenia, as well as increased susceptibility to osteoporosis-related fractures	[23]
	Pregnant Wistar rats were administrated intragastrically with CAF (120 mg/kg/day) on gestational days 9–20	Histone	PCE decreased the H3K9ac level of 11β-HSD2 in fetal bone tissue which maintained to the adult via the increased fetal serum corticosterone concentration that promoted GR activation and recruited HDAC11 to decrease the H3K9ac	Associated with bone development dysplasia and increased osteoporosis susceptibility in male adult offspring	[24]
Ovary	From GD9 to GD20, the pregnant Wistar rats were intragastrically treated with 30 or 120 mg/kg/day CAF	Histone	PCE inhibited H3K27ac at the IGF-1 promoter region in fetal ovary through GR/HDAC10 signaling which was due to PCE-induced GC exposure	Induced ovarian dysfunction in adulthood	[25]
Cardiovascular system	Mice of a mixed background (129/OlaHsd/C57BL) were mated, and pregnant mice were injected 20 mg/kg of CAF intraperitoneally at embryonic day 8.5. In adult offspring left ventricles: analysis of the number of differentially methylated regions (DMRs) within the genomic DNA between CAF- and A1AR-dependent (veh+/+ vs. caff+/+), CAF-dependent and A1AR-independent (veh−/− vs. caff−/−)	DNA	In veh+/+ vs. caff+/+ group: 4896 regions were hypermethylated and 2823 regions were hypomethylated In veh−/− vs. caff−/− group: 1024 regions were hypermethylated and 1757 regions were hypomethylated There were 103 miRNA regions between veh+/+ and caff+/+ that were significantly differentially methylated	Related to the development of the heart	[26]
	Several DMRs at different gene loci related to cardiac hypertrophy, including Mef2c, Tnnt2, Myh6, Myh7, and Gata4 and body weight Ins2 were used for bisulfite sequencing in veh+/+ vs. caff+/+ comparison		Mef2c and Ins2, which were both hypermethylated, and Myh6, which was hypomethylated, in caff+/+ matched the DNA methylation array results Gata4 and Myh7 showed no difference by BS-seq in caff+/+ even though they were both hypermethylated in the DNA methylation array results Tnnt2 was hypomethylated in the caff+/+ group in the array but hypermethylated by BS-seq		
	DNA methylation level throughout the whole genome was detected in adult offspring left ventricles		CAF treatment caused a 26% decrease in global DNA methylation in A1AR+/+ hearts, but no change in the level of DNA methylation in the A1AR+/− hearts No significant change in global DNA hydroxymethylation was detected in either A1AR+/+ or A1AR+/− adult hearts following in utero CAF exposure		
	CD-1 mice were injected intraperitoneally from embryonic day 6.5 to 9.5 with 20 mg/kg/day CAF, and global DNA methylation level was examined in embryonic heart ventricles at 24 h after the last CAF dose	DNA and ncRNA	Global cardiac DNA methylation was significantly increased by CAF which was associated with the change of DNA methylation enzymes	Related to cardiovascular development and related diseases	[27]
	CD-1 mice were injected intraperitoneally from embryonic day 6.5 to 9.5 or from embryonic day 6.5 to 10.5 with 10, 20, and 60 mg/kg/day CAF, and gene expression was examined in embryonic heart ventricles at either 24 or 2 h after the last CAF dose		Treatment from embryonic day 6.5 to 10.5: the expression of miR-208a was increased, and the expression of miR-208b and miR-499 were decreased Treatment from embryonic day 6.5 to 9.5: miR-208a was consistently upregulated and miR-499 was consistently downregulated, but miR-208b did not change		
	Primary embryonic cardiomyocytes were treated with increasing concentrations of CAF for 48 h		CAF reduced the expression of miR-208a, and increased the expression of miR-208b and miR-499		
	HL-1 cardiomyocytes were treated with increasing concentrations of CAF for 48 h		Increased the expression of some cardiac miRNAs, including miR-208a, miR-208b, and miR-499		
Colon carcinoma	Caco-2 human colon carcinoma cells were treated with 5% coffee extract for the indicated times Cells were treated with 0–5% coffee extracts for 3 h	ncRNA	The expression of miR-30c and miR-96 in Caco-2 cells was induced 3 h following the treatment and gradually decreased to basal levels over time. The induction of miRNAs occurred in a dose-dependent manner Coffee may upregulate miR-30c in Caco-2 cells by activating NF-κB	Prevented the malignant growth of colon carcinoma cells	[30]
	RKO human colon cancer cells (ATCC; CRL-2577) were cultured in the presence of 1000 µM CGA for 24, 48, and 72 h	ncRNA	CGA reduced the expression levels of the oncogene-miR-31	Related to the decreased colon cancer cell proliferation	[31]
	DMH/DCA mouse model was treated with CAF and CGA (50 and 25 mg/kg intragastrically) for 10 weeks	ncRNA	CAF + CGA intervention significantly increased the expression of two miRNAs (miR-451a and miR-151-5p), while it downregulated four miRNAs (miR-21a-5p, miR-143-3p, miR-26b-5p, and miR-223-3p) compared to the DMH/DCA group in colon	Attenuated preneoplastic ACF development	[32]
Leukemia	Jurkat and HL-60 cells were treated with 100 μM CGA or CaA for 72 h, and the global DNA methylation levels were analyzed	DNA	CGA, but not CaA, decreased the levels of 5-mC in Jurkat cells significantly; in HL-60 cells, neither phenolic acid altered the levels of 5-mC	Showed benefits against hematological malignances whose pathogenic processes involve impairment of DNA methylation	[34]
Breast cancer	The MCF-7 human breast cancer cells were treated with 1, 5, 20, or 50 μM of CaA or CGA for 8 days The MDA-MB-231 human breast cancer cells, were treated with 0.2, 1, 5, or 20 μM of CaA or CGA for 3 days The T-47D human breast cancer cells were treated with 20 or 50 μM CaA or CGA for 2 days	DNA	Treatment of MCF-7 human breast cancer cells demethylated the hypermethylated RARb gene in a dose-dependent manner, which was similar in MDA-MB-231 cells In MCF-7 cells and MDA-MB-231cells, the methylation-specific band for the RARb gene after treatment was decreased, while the unmethylation-specific band of the RARb gene was increased In T-47D cells, treatment with CGA increased the unmethylation-specific band at the promoter region of the p16 gene, while changes in the methylation-specific band of this gene were less pronounced	----	[35]
Cancer risk	The human HCC cells (MHCC97H) were subcutaneously injected into the right armpit of the BALB/c nude mice to establish tumors, and CaA (0 or 10 mg/kg) was administered intraperitoneally twice per week for 11 weeks MHCC97H cells were treated with 0 or 20 μM CaA for 48 h	DNA and ncRNA	CaA enhanced the expression of miR-148a by demethylation in vitro and in vivo	Attenuated the CSC-like properties	[36]
	Female CBA/Ca mice treated with DMBA received a coffee (*Coffea arabica*) extract for 2 weeks (30 mg/day/animal, up to 150 mL)	ncRNA	Coffee extract significant decreased the expression of miR-9-3 in the liver, the expression of miR-9-3 and miR-134 in the spleen, the expression of miR-9-3, miR-124-1, and miR-134 in the kidney	Revealed the chemopreventive effects of coffee in cancer risk	[37]
Cataract	Young CD-1 mice were fed with diet containing 25% galactose with 1% CAF for 7 days	ncRNA	Adding CAF to the galactose diet significantly annulled the elevation of some miRNAs caused by galactose in lenses	Attenuated the cataract formation	[45]
Glaucoma	Ocular hypertension ischemia-reperfusion (I/R) glaucoma mouse model was intragastrically administered CGA at a dose of 10 mg/kg, 20 mg/kg, or 30 mg/kg every day for 2 weeks H_2_O_2_-induced retinal ganglion cell (RGC-5) was treated with CGA at the concentration of 25, 50, or 100 μM for 6 h	ncRNA	lncRNA-TUG1 expression was upregulated after the treatment of CGA in H_2_O_2_-induced RGC-5 cells and mouse retinal tissue	Relieved retinal injury in glaucoma mouse model	[46]
Pathogen infection	MH-S, MLE-12, and primary murine bone marrow-derived macrophages (BMDMs) were treated with CAF (0.1, 0.25, 0.5, and 1 mM for 24 h). Mice were treated with 50 mg/kg CAF for indicated time	ncRNA	miR-301b, -301a, and -15a were inhibited in a dose- and time-dependent manner in cells and the lung tissue from mice treated with CAF	Alleviated lung injury and decreased the susceptibility to bacterial infection	[47]
	Mice were infected with *Pseudomonas aeruginosa* (1 × 10^7^ colony-forming units) for 24 h, and CAF (50 mg/kg) was injected at indicated time (pretreatment strategy or post-infection treatment)		The expression of miR-301b in the lung was decreased		
	After CAF pretreatment for 24 h, LPS (100 ng/mL) was added for another 6 h incubation with MH-S, MLE-12, and BMDM cells		Led to the strongest inhibition in LPS-induced miR-301b through negative regulation of cAMP/PKA/NF-κB signaling		
	LPS-induced ALI mouse was continuously given CGA (100 mg/kg) by gavage for 7 days	ncRNA	The expression levels of miR-223 in lung tissues were significantly increased	Alleviated pathological damage of lung tissues	[48]
	LX2 cells were cultured in the presence of CGA at 80 μg/mL, 40 μg/mL, and 20 μg/mL for 24 h and treated with IL-13 from 5 to 100 ng/mL for 6 h Male BALB/c mice infected with 25 ± 5 *Schistosoma japonicum* cercaria were administered praziquantel (500 mg/kg) for 5 days, and then treated with CGA (5, 10, 20 mg/kg) for 4 weeks	ncRNA	CGA could inhibit the expression of miR-21 in LX2 cells and liver	Reduced the degree of liver fibrosis in pathological manifestations	[49]
	ST-induced mouse enteritis model (female C57BL/6 mice) was treated with different concentrations of CGA (10, 60, and 120 mg/kg/day) by gavage for 7 days	ncRNA	The GAS5 expression in the intestinal tissue was upregulated significantly with a high concentration of CGA The expression of miR-23a in intestinal tissues was significantly downregulated	Reduced intestinal damage caused by ST	[50]
Brain	Male UChB rats were treated with a bottle containing the ethanol solution (1:10), along with CAF in a concentration of 3 g/L from the 95th to 150th day	ncRNA	The miR-146a-5p, -155-5p, -132-3p, and -126-3p expressions were lowered in the cerebellum The miR-126-3p and miR-132-3p levels were elevated in the plasma miR-155-5p expression was decreased in the plasma	CAF had a neuroprotective effect against ethanol on the cerebellar tissue	[53]
	Mouse microglia (BV2) and mouse hippocampal neurons (HT-22) were treated with CGA (5, 10, and 20 μM) for 24 h beforehand and then with OGD for 6 h	ncRNA	CGA heightened the profiles of MIR497HG and restricted miR-29b-3p expression in OGD-elicited cells	Reduced the hypoxic ischemia reperfusion damage in cells	[54]
	CUMS model group (male Wistar rats) was treated with CaA intraperitoneally at doses of 10 and 50 mg/kg at 9:00 a.m. every day for 4 weeks	DNA	In the hippocampus, the levels of 5mC were slightly lowered, and the levels of 5hmC were slightly increased in the CaA-treated group In the prefrontal cortex, no obvious changes in global 5mC and 5hmC levels was observed following CaA treatment CaA could have led to the above epigenetic change by inducing different Dnmt1/Dnmt3a and Tet1/Tet2 mRNA levels in the hippocampus and prefrontal cortex	Exerted a slight antidepressant-like effect	[55]
Liver	Hepa 1–6 cells were incubated for 24 h with 2.5 × 10^−4^% of CPP	ncRNA	CPP increased miR-122 levels in Hepa 1–6 cells	Related to suppression of body fat accumulation	[57]
	From GD9 to GD20, pregnant rats were administered intragastrically with different doses of CAF (30, 60, and 120 mg/kg/day)	Histone	PCE-induced high GC levels enhanced histone modifications (H3K14ac and H3K27ac) at the promoter regions of SREBP-1c and FASN via activation of the GR-C/EBPα-SIRT1 pathway in female fetal rats	Induced hepatic lipid accumulation and increased the susceptibility to NAFLD in PCE adult female rats	[58]
	From GD9 to GD20, pregnant Wistar rats were administered 30, 60, and 120 mg/kg/day CAF intragastrically	Histone	PCE increased the hepatic H3K9ac and H3K14ac at the promoter of HMGCR in the high dose PCE group induced by the increased GR-C/EBPα signaling via high levels of GC in utero	Induced hypercholesterolemia in adult female offspring	[59]
	From GD9 to GD20, the Wistar rats in PCE groups were administered 30, 60, and 120 mg/kg∙day CAF intragastrically Human fetal hepatic cells (L02 cell line) were treated with 0.1, 1, 10, and 100 μM CAF for 24 h	Histone	H3K14ac and H3K27ac at the promoter regions of hepatic cholesterol synthesis genes (Srebf2, Hmgcr, and Hmgcs1) were increased by inhibiting the SIRT1 expression in fetal livers of male offspring which was due to the CAF-induced inhibition of A2AR/cAMP/PKA pathway H3K14ac and H3K27ac at the promoter of the SREBF2, HMGCR, and HMGCS1 were increased in L02 cells due to the CAF-induced inhibition of A2AR/cAMP/PKA pathway	Induced the susceptibility to hypercholesterolemia and related cardiovascular and cerebrovascular diseases in male adult offspring	[60]
	LX2 cells were treated with CGA at various concentrations (20 μg/mL, 40 μg/mL, and 80 μg/mL) for 24 h. For the last 6 h, TGF-β1 (10 ng/mL) was added	ncRNA	The levels of miR-21 were decreased both in liver tissue and in LX2 cells	Lessened the degree of liver fibrosis in the pathological manifestation	[61]
	The CCl_4_-induced fibrosis rat model (male Sprague-Dawley rats) was administered CGA at 15 mg/kg, 30 mg/kg, or 60 mg/kg for 4 consecutive weeks				
Kidney	Diabetic kidney disease (DKD) model male Wistar rats were treated with CaA (40 mg/kg body weight/day orally for 4 weeks) or pretreated with CaA after induction of diabetes for 4 weeks	ncRNA	CaA inhibited miR-133b, -342, and -30a expressions in DN rats	Improved DN	[66]
	Diabetes mellitus adult male Albino Wistar rat model induced by STZ was treated with CaA (40 mg/kg/day) for 8 weeks	ncRNA	CaA treatment significantly reduced the level of renal miR-636 expression compared to DN group in the kidneys	CaA effectively ameliorated renal damage in diabetic rats	[67]
	From GD9 to GD20, the pregnant Wistar rats were intragastrically treated with 30, 60, and 120 mg/kg/day CAF	Histone	PCE reduced the H3K9ac level at the KLF4 promoter region in the fetal kidney via the increased fetal serum corticosterone concentration that promoted GR activation and recruited HDAC7 to decrease the H3K9ac	Induced podocyte dysplasia in utero and podocyte phenotypic damage in adulthood in male offspring that contributes to glomerular diseases such as glomerulosclerosis	[68]
Nuclear reprogramming	Somatic cell nuclear transfer embryos fused with porcine fetal fibroblasts at the G0/G1 stage and enucleated oocytes were treated with 5 mM CAF for 2.5 h	DNA	Induced efficient demethylation in the NTs and a DNA methylation pattern at the four-cell stage that was similar to that for in vitro fertilized (IVF) embryo	Resulted in nuclear reprogramming	[75]
Lifespan	Honeybees were treated with the syrup supplemented with CAF at the concentration of 5 μg/mL from 1 day old DNA was extracted from the head and thorax for detection	DNA	From 1 to 3 days old, CAF led to little change in DNA methylation levels; however, CAF significantly decreased DNA methylation levels in older bees	Extended the lifespan and increased resistance to Nosema	[76]

## Data Availability

Not applicable.

## Abbreviations

ACE	Angiotensin converting enzyme
ACF	Aberrant crypt foci
AD	Alzheimer’s disease
AKI	Acute kidney injury
ALI	Acute lung injury
ALL	Acute lymphoblastic leukemia
CA or CaA	Caffeic acid
CAF	Caffeine
CGA	Chlorogenic acid
CPP	Coffee polyphenols
CRH	Corticotrophin-releasing hormone
CSCs	Cancer stem cells
CUMS	Chronic unpredictable mild stress
DMBA	7,12-dimethylbenz[a]anthracene
DMH/DCA	1,2-dimethylhydrazine/deoxycholic acid
DMRs	Differentially methylated regions
DN	Diabetic nephropathy
GC	Glucocorticoid
GD	Gestational day
GR	Glucocorticoid receptor
HMGCR	3-hydroxy-3-methylglutaryl-CoA reductase
HPA	Hypothalamic–pituitary–adrenal
IGF-1	Insulin-like growth factor 1
IUGR	Intrauterine growth retardation
KLF4	Kruppel-like factor 4
LINE-1	Long interspersed element-1
LPS	Lipopolysaccharide
MDD	Major depressive disorder
MEF	Myocyte enhancer factor
NAFLD	Nonalcoholic fatty liver disease
NF-κB	Nuclear factor-kappa B
NLRP3	Nucleotide-binding oligomerization domain-like receptor protein 3
Nrf2	Nuclear factor erythroid-2 related factor 2
NTs	Nuclear transfer embryos
OGD	Oxygen and glucose deprivation
PCE	Prenatal caffeine exposure
PD	Parkinson’s disease
RAS	Renin angiotensin system
RGC	Retinal ganglion cell
SF-1	Steroidogenic factor-1
SIRT1	Sirtuin-1
SR-BI	Scavenger receptor class B type I
SREBP	Sterol regulatory element-binding protein
ST	*Salmonella typhimurium*
StAR	Steroidogenic acute regulatory protein
STZ	Streptozotocin
TGFβR1	Transforming growth factor beta receptor 1
11β-HSD-2	11β-hydroxysteroid dehydrogenase-2
